# Effect of the TGF-β/BMP Signaling Pathway on the Proliferation of Yak Pulmonary Artery Smooth Muscle Cells under Hypoxic Conditions

**DOI:** 10.3390/ani14142072

**Published:** 2024-07-15

**Authors:** Junfeng He, Kejin Wang, Biao Wang, Yan Cui, Qian Zhang

**Affiliations:** 1 Laboratory of Animal Anatomy & Tissue Embryology, Department of Basic Veterinary Medicine, Faculty of Veterinary Medicine, Gansu Agricultural University, Lanzhou 730070, China; wangb0503@163.com (B.W.); cuiyan@gsau.edu.cn (Y.C.); zq880204@126.com (Q.Z.); 2 Gansu Provincial Center for Disease Control and Prevention, Lanzhou 730070, China; www.wkj@outlook.com; 3 Gansu Province Livestock Embryo Engineering Research Center, Department of Clinical Veterinary Medicine, Faculty of Veterinary Medicine, Gansu Agricultural University, Lanzhou 730070, China

**Keywords:** yak, hypoxia, pulmonary artery smooth muscle cells, proliferation

## Abstract

**Simple Summary:**

The yak (*Bos grunniens*) is a unique breed in the pastoral areas of the Qinghai–Tibet Plateau. It has the ability to adapt to the extremely low oxygen environment in the plateau and is one of the important livestock breeds in the plateau. Yaks can live in an extremely low-oxygen environment for a long time and avoid pulmonary hypertension caused by hypoxia. However, little is known about the molecular regulatory mechanisms of how yaks avoid pulmonary hypertension. In this study, the dynamic regulation of the TGF-β/BMP signal in yak pulmonary artery smooth muscle cells has positive significance in maintaining homeostasis and adapting to hypoxic environments.

**Abstract:**

To survive in low-oxygen environments, yaks effectively avoid hypoxia-induced pulmonary arterial hypertension through vascular remodeling. The TGF-β/BMP signaling pathway plays a key role in maintaining the homeostasis of pulmonary artery smooth muscle cells (PASMCs). However, little is known about the molecular regulatory mechanisms by which the TGF-β/BMP signaling pathway contributes to the proliferation of yak PASMCs. In this study, yak PASMCs were cultured in vitro, and a hypoxia model was constructed to investigate the effect of TGFβ/BMP signaling on yak PASMC proliferation. Hypoxia treatment increased the proliferation of yak PASMCs significantly. As the duration of hypoxia increased, the expression levels of TGF-β1 and the phosphorylation levels of Smad2/3 were upregulated significantly. The BMP signaling pathway was transiently activated by hypoxia, with increases in BMPR2 expression and Smad1/5 phosphorylation, and these changes were gradually reversed with prolonged hypoxia exposure. In addition, exogenous TGF-β1 activated the TGF-β signaling pathway, increased the phosphorylation levels of the downstream proteins Smad2 and Smad3, and increased the proliferation and migration rates of yak PASMCs significantly. Finally, treatment with noggin (an inhibitor of BMP signaling) significantly reduced BMPR2 protein expression levels and Smad1/5 phosphorylation levels and increased yak PASMC proliferation and migration rates. In summary, these results revealed that under hypoxic conditions, the dynamic regulation of the TGF-β/BMP signaling pathway promotes the proliferation of yak PASMCs.

## 1. Introduction

The yak (*Bos grunniens*) is an exceptional bovine breed indigenous to the pastoral regions of the Tibetan Plateau. Through long-term natural selection, yaks have developed remarkable adaptations to high altitudes, extreme cold, and low-oxygen environments [1]. In particular, during long-term adaptation to hypoxia, yaks have developed unique lung structures and functions [2], allowing them to maintain a low pulmonary artery pressure and a reduced pulmonary vasoconstriction response [3], preventing hypoxic pulmonary hypertension (HPH) [4]. Accordingly, the yak has gained attention as a natural large mammalian model for resistance to HPH in hypoxic environments. Studies have shown that cattle breeds from lower altitudes develop severe HPH when moved to the habitats of yaks, while yaks can survive for long periods in extreme hypoxic environments without damage to their lungs [5]. The etiology of HPH is multifactorial, mainly involving pulmonary vasoconstriction and medial thickening of the pulmonary vessels due to alveolar hypoxia [6,7]. PASMC proliferation is usually associated with pulmonary vascular remodeling in non-high-altitude mammals [8]; however, in mammals that live at high altitudes, like yaks, PASMC proliferation is essential for lung development and adaptation to hypoxic environments [4,9]. Therefore, it is necessary to explore the mechanisms by which yaks adapt to the hypoxic environment of the Tibetan Plateau and how they circumvent HPH.

Zhang et al. [10] found that HIF-1α expression in yak lung tissues increases as the duration of exposure to hypoxic conditions increases. Another comparative proteomic study of PASMCs cultured under normoxic and hypoxic conditions showed that Smad2 is upregulated significantly under hypoxia [11]. Cell proliferation plays a pivotal role in the growth and development of animals [12]. The dysregulation of transforming growth factors β1 (TGF-β1)/bone morphogenetic protein (BMP) signaling is involved in hypoxia-induced pulmonary vascular remodeling [13]. However, studies related to the dysregulation of the TGF-β/BMP signaling pathway are largely based on patients with HPH or animal models, and the mechanisms by which signaling crosstalk between the TGF-β and BMP pathways, especially in yak PASMCs, contribute to the maintenance of vascular function are not fully understood. In this study, yak PASMCs, a natural cell model resistant to hypoxic pulmonary hypertension, were used to investigate the expression patterns of TGF-β1/BMP signaling components under hypoxic conditions and their regulatory effects on PASMC proliferation. The results of this study provide insight into hypoxia adaptation in yaks and suggest that balancing the TGF-β/BMP signaling pathway could be an effective therapeutic approach for HPH.

## 2. Materials and Methods

### 2.1. Sample Collection

Lung tissues were collected from 6-month-old yaks (*n* = 3) residing at altitudes of 3000 m in the Qinghai area. Healthy juvenile yaks were euthanized by arterial bleeding, and their cardiopulmonary organs were removed and placed in a sterile tray. The organs were repeatedly rinsed with saline containing 2% penicillin and streptomycin and then quickly transferred to a sterile workbench. All experimental procedures were conducted following the guidelines set by the Ministry of Science and Technology of the People’s Republic of China (approval number: 2006-398). This study was approved by the Animal Ethics Committee of Gansu Agricultural University (approval number: GSAU-Eth-VMC-2023-012).

### 2.2. Isolation and Culture of Yak PASMCs

(1) Under sterile conditions, the pulmonary trunk and its branches were progressively peeled away from the right ventricle; the large trunk was discarded, and the smaller branches were retained.

(2) The dissected pulmonary artery walls were flattened, and sterile tweezers and scissors were used to scrape away the inner and outer membranes gently and repeatedly. The samples were rinsed repeatedly with saline containing 2% penicillin and streptomycin until the rinse fluid was clear.

(3) The clean arterial samples were chopped into 1 mm^3^ pieces, transferred to a 15 mL centrifuge tube, and rinsed twice with saline containing 2% penicillin and streptomycin.

(4) A pre-warmed mixture of 1 mg/mL collagenase I (C0130; Sigma, St. Louis, MO, USA) and 1 mg/mL collagenase II (C6885; Sigma, St. Louis, MO, USA) was added, followed by incubation on a 37 °C shaker for 4–5 h until the tissue turned into a flocculent mass. After centrifugation at 500 *g* for 10 min, the supernatant was discarded, and the pellet was resuspended in DMEM/F12 (12400024; Gibco, NY, USA) complete medium containing 20% FBS (10099141C; Gibco, NY, USA) and 1% penicillin–streptomycin, followed by pipetting to create a cell suspension. The suspension was added to a T25 cell culture flask and cultured in an incubator at 37 °C and 5% CO_2_.

### 2.3. Purification of Primary Yak PASMCs

According to the cell morphology and growth characteristics of fibroblasts and PASMCs, the differential adhesion method was used to purify yak PASMCs. When the primary cells reached approximately 90% confluence, they were passaged. First, the cells were rinsed twice with PBS. After removing the PBS, 1 mL of 0.25% trypsin (27250018; Gibco, NY, USA) was added. The cells were then placed in a 37 °C, 5% CO_2_ incubator for 2 min. After the cells detached, 2 mL of complete culture medium was added to terminate digestion. The cells were resuspended, added to a culture flask, and placed in the incubator for 15 min. Most of the fibroblasts adhered first; then, the cell suspension was transferred to a new culture flask. This step was repeated 1–2 times for purification.

### 2.4. Determination of Growth Curves of PASMCs

After three passages and digestion, the cells were seeded at a density of 8000 cells per well in a 96-well plate. At fixed time points each day, three wells were treated with trypsin for digestion to prepare a cell suspension. Then, 10 μL of the cell suspension was dropped onto a cell counting chamber slide. Cells in four large squares were counted under a microscope (100×), and this process was repeated daily for 7 days. The total number of cells per mL was calculated as the total number of cells in the four squares divided by 4, multiplied by 10^4^ and the dilution factor. Based on the cell counts, the growth curve for yak PASMCs was generated.

### 2.5. Immunofluorescence Staining

(1) Cells in the logarithmic growth phase were seeded at an appropriate density in culture dishes containing coverslips. Samples were incubated in a 5% CO_2_, 37 °C incubator until reaching approximately 60% confluence. The cell medium was discarded. The samples were washed twice with PBS and then fixed with 2% paraformaldehyde for 1 h.

(2) After the fixative was discarded, the samples were washed three times with PBS, supplemented with 0.1% Triton X-100 (P0096; Beyotime, Shanghai, China), incubated at 25 °C for 20 min, and washed three times with PBS.

(3) The samples were treated with 5% BSA (SW3015; Solarbio, Beijing, China) and incubated at 25 °C for 1 h to block non-specific sites.

(4) After discarding the 5% BSA, the liquid at the edge of the slide was aspirated, diluted primary antibodies against α-SMA (AF0048; Affinity, Changzhou, Jiangsu, China), calponin (bs-0095R; Bioss, Beijing, China), and CD31 (ab119339; Abcam, Cambridge, MA, USA) were added dropwise, and PBS as a negative control was added dropwise, followed by incubation at 4 °C overnight.

(5) The samples were washed three times with PBS, the fluorescent secondary antibody was added (8889s; Cell Signaling Technology, Boston, MA, USA), and the samples were incubated for 1 h at 25 °C in the dark.

(6) The samples were washed three times with PBS, followed by the addition of DAPI staining solution dropwise and incubation in the dark for 5 min.

(7) After three additional washes with PBS, an anti-fade mounting medium was added dropwise to the slides.

(8) A fluorescence microscope imaging system was used for observations and imaging.

### 2.6. Establishment of a Hypoxia Model of Yak PASMCs

To simulate hypoxic conditions, yak PASMCs were incubated in an environment containing 5% O_2_. The oxygen concentration was controlled by injecting nitrogen gas into a tri-gas incubator. In the normoxic control group, yak PASMCs were incubated in a standard incubator at 37 °C with 21% O_2_ and 5% CO_2_. The hypoxic group was maintained in a tri-gas incubator at 37 °C with 5% CO_2_ and 5% O_2_.

### 2.7. Cell Proliferation and Scratch Test

Yak PASMCs were seeded at a density of 10^4^ cells per well in a 96-well plate. Various concentrations of TGF-β1 recombinant protein (HY-P7118; MedChemExpress, Middlesex County, NJ, USA) and a noggin inhibitor (HY-P70558; MedChemExpress, Middlesex County, NJ, USA) were added to the cells (except in the control group, where no additives were used). After incubation for 24 h in the incubator, according to the instructions provided with the CCK8 assay kit (HY-K0301; MedChemExpress, Middlesex County, NJ, USA), 10 µL of CCK8 solution was added to each well, and incubation was continued for another 2 h. The optical density (OD) of the cells at 450 nm was measured and recorded using a microplate reader (Thermo Fisher Scientific, Waltham, MA, USA).

A scratch assay was used to assess the migration of yak PASMCs. A ruler was used to draw straight, horizontal lines at the bottom of a 6-well plate, spaced 1.5 cm apart. Cells were seeded at a density of 2 × 10^4^ cells per well. When the cell density reached approximately 80%, the cells were treated with a basic medium for 12 h. A 200 μL sterile pipette tip was then used to make a vertical scratch on the cell layer in the 6-well plate. The detached cells were rinsed with PBS. For the control group, a medium containing 0.2% FBS was used. For the experimental groups, 5 ng/mL TGF-β1 recombinant protein and 100 ng/mL noggin inhibitor were added, followed by incubation for 24 h. Cell migration was observed under a microscope.

### 2.8. Extraction of Total Cellular Proteins

Petri dishes containing cells under different oxygen concentrations were removed from the incubator, and the culture medium was quickly discarded. The cells were rinsed three times with pre-cooled PBS. Then, 200 μL of pre-cooled RAPI cell lysis buffer (containing 2 μL PMSF and 2 μL phosphatase inhibitor) (R0010, Solarbio, Beijing, China) was added, and the samples were placed on ice for 10 min to lyse the cells. A pipette was used repeatedly until the cells were fully lysed, and the lysate was added to a 1.5 mL EP tube. After centrifugation, the supernatant was retained. The cell lysate was mixed with 4× protein sampling buffer at a ratio of 3:1. The mixture was placed in a 100 °C constant temperature metal bath for 10 min, cooled, and stored at −20 °C for later use.

### 2.9. Western Blotting

After the protein samples were separated by 12% polyacrylamide gel electrophoresis, the proteins were transferred to a 0.22 µm PVDF membrane. The membrane was blocked at room temperature with 50 g·L^−1^ non-fat dry milk powder for 3 h. Then, the samples were incubated overnight at 4 °C with primary antibodies for HIF-1α (1:1000; ab16066, Abcam, Cambridge, MA, USA), HIF-2α (1:800; AF6362, Affinity, Jiangsu, China), TGF-β1 (1:500; bs-0086R, Bioss, Beijing, China), Smad2 (1:500; bs-0718R, Bioss, Beijing, China), Smad3 (1:500; bs-8853R, Bioss, Beijing, China), phosphorylated Smad2 (P-Smad2) (1:400; bs-8853R, Bioss, Beijing, China), phosphorylated Smad3 (P-Smad3) (1:400; bs-3425R, Bioss, Beijing, China), BMPR2 (1:500, bs-4237R; Bioss, Beijing, China), Smad1/5 (1:500; AF0614, Affinity, Jiangsu, China), phosphorylated Smad1/5 (P-Smad1/5) (1:400; bs-3418R, Bioss, Beijing, China), PCNA (1:500; bs-2006R, Bioss, Beijing, China), and BCL-2 (1:500; bs-0032R, Bioss, Beijing, China). The membrane was washed for 1 h with PBST (using TBST phosphorylated antibodies) and incubated with the secondary antibody (1:3000; bs-0295G-HRP, Bioss, Beijing, China) on a shaker at 25 °C for 1 h. ECL (Enhanced Chemi-luminescence) detection reagent was applied, and the samples were scanned using a chemiluminescence detection system. β-actin was used as a loading control, and densitometry was performed using ImageJ (1.8.0).

### 2.10. Data Analysis

Data were analyzed using GraphPad Prism 6. Results are presented as the mean ± standard error of the mean (SEM). Groups were compared using unpaired *t*-tests or one-way analysis of variance (ANOVA). Values of *p* < 0.05 were considered significant.

## 3. Results

### 3.1. Isolation, Culture, and Characterization of Yak PASMCs In Vitro

**Figure 1:**
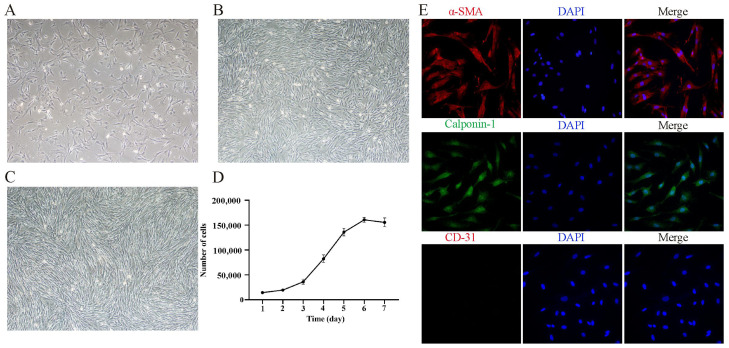
Isolation, culture, and characterization of yak PASMCs. (**A**) Morphological characteristics of primary yak PASMCs on day 3 of culture. (**B**) Morphological characteristics of primary yak PASMCs on day 4 of culture. (**C**) Morphological characteristics of primary yak PASMCs on day 6 of culture, showing the typical “hill-and-valley” pattern; magnification: ×200. (**D**) Growth curve of yak PASMCs. (**E**) Immunofluorescence identification of yak PASMCs; α-SMA is red; Calponin-1 is green; CD-31 is red; and DAPI is blue. Magnification: ×200.

Primary yak PASMCs were isolated and cultured using an enzymatic digestion method. By the third day of culture, cells began to adhere and appeared elongated (Figure 1A). By the fourth day, approximately 85% of the cells were adherent and growing (Figure 1B). By the sixth day, cells grew in clusters and gradually formed a typical “hill-and-valley” pattern (Figure 1C). The growth curves of yak PASMCs was plotted based on cell count data, showing slow growth on days 1–3, representing the initial growth phase, a logarithmic growth phase from days 3–6 with significant proliferation, and a stabilization phase on day 7 (Figure 1D). In addition, we identified yak PASMCs by immunofluorescence staining. Positive expression of the smooth muscle cell marker proteins α-SMA and Calponin-1 and negative expression of the endothelial cell marker protein CD31 (Figure 1E) indicated successful isolation and cultivation of primary yak PASMCs.

### 3.2. Effect of Hypoxia on BMP/TGF-β Signaling Pathway-Related Protein Expression and Proliferation in Yak PASMCs

**Figure 2:**
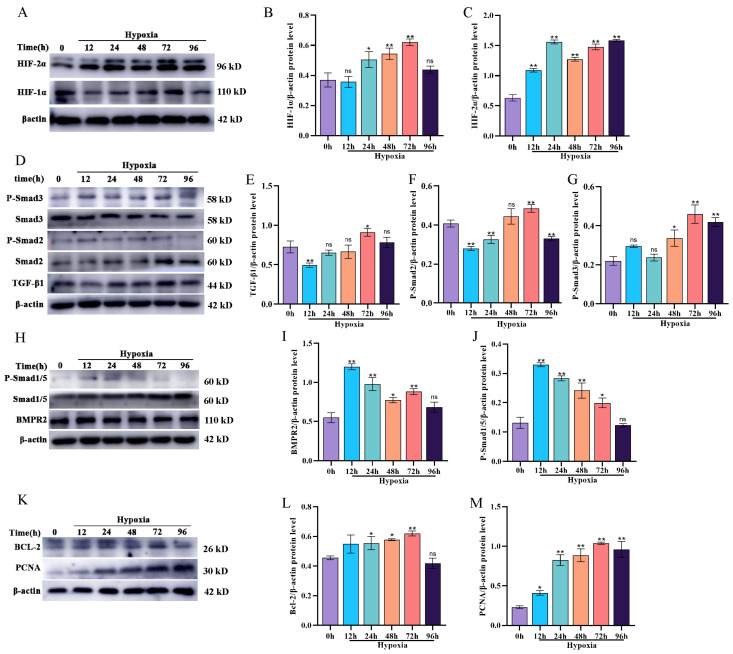
Impact of hypoxia on BMP/TGF-β signaling pathway-related protein expression and proliferation in yak PASMCs. (**A**–**C**) Western blot analysis of HIF-1α and HIF-2α protein expression under hypoxic conditions, with a densitometric analysis performed using ImageJ. (**D**–**G**) Western blot analysis of TGF-β/Smad2/3 signaling pathway-related protein expression under hypoxia conditions, with a densitometric analysis performed using ImageJ. (**H**–**J**) Western blot analysis of BMP/Smad1/5 signaling pathway-related protein expression under hypoxic conditions, with a densitometric analysis performed using ImageJ. (**K**–**M**) Western blot analysis of BCL-2 and PCNA protein expression levels under hypoxic conditions, with a densitometric analysis performed using ImageJ. ** p < 0.01, * 0.01 < *p* < 0.05, ns indicates insignificant difference.

To investigate the impact of hypoxia on BMP/TGF-β signaling pathway-related protein expression and proliferation in yak PASMCs, 21% O_2_ was used for the control group and 5% O_2_ was used for the hypoxic treatment group. PASMCs were cultured for 12, 24, 48, 72, and 96 h under these conditions. Initially, the expression levels of key regulatory factors in the response to hypoxia, HIF-1α and HIF-2α, were detected by Western blotting. The levels of both HIF-1α and HIF-2α increased significantly (*p* < 0.05) after 24 h of exposure to hypoxia (Figure 2A–C), confirming the reliability of the yak PASMC response to hypoxia. Furthermore, the expression levels of proteins related to the BMP/TGF-β signaling pathway were analyzed. Compared with levels in the normoxic group, the expression levels of TGF-β1, P-Smad2, and P-Smad3 were lower after 12 h of hypoxia (Figure 2D–G) but increased over time, peaking at 72 h. Conversely, the expression levels of proteins in the BMP signaling pathway showed the opposite trends to those in the TGF-β signaling pathway. The levels of the BMP signaling pathway-related proteins BMPR2 and P-Smad1/5 were significantly higher in the hypoxia group than in the normoxic group and decreased gradually as the duration of hypoxia increased, approaching levels in the normoxic group at 96 h (Figure 2H–J). These results indicate that hypoxia activates the TGF-β signaling pathway in yak PASMCs. The BMP signaling pathway is transiently activated by hypoxia, and this trend is gradually reversed with prolonged hypoxia exposure. In addition, Western blotting revealed that hypoxia promotes proliferation and inhibits apoptosis in yak PASMCs (Figure 2K–M).

### 3.3. Selection of Drug Concentrations for TGF-β1 Recombinant Protein and Noggin

**Figure 3:**
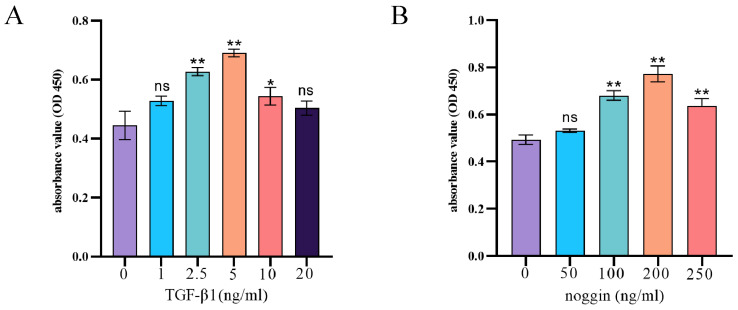
Optimization of concentrations of TGF-β1 recombinant protein and noggin. (**A**) CCK8 assay of the cytotoxicity of TGF-β1 recombinant protein on yak PASMCs. (**B**) CCK8 assay of the cytotoxicity of noggin on yak PASMCs. ** p < 0.01, * 0.01 < *p* < 0.05, ns indicates insignificant differences.

To evaluate the effects of exogenous TGF-β1 and the BMP signaling pathway inhibitor noggin on yak PASMCs, we used a CCK8 kit to assess the cytotoxicity of various concentrations. Briefly, various concentrations of exogenous TGF-β1 and noggin were administered to PASMCs for 24 h. The CCK8 assay results indicated that 2.5–10 ng/mL exogenous TGF-β1 recombinant protein significantly increased the proliferation of PASMCs over that of untreated cells (Figure 3A), and 100–200 ng/mL noggin significantly increased the proliferation of yak PASMCs (Figure 3B). Therefore, these concentrations were used for subsequent analyses.

### 3.4. TGF-β1 Activates the TGF-β Signaling Pathway and Promotes the Proliferation of Yak PASMCs

**Figure 4:**
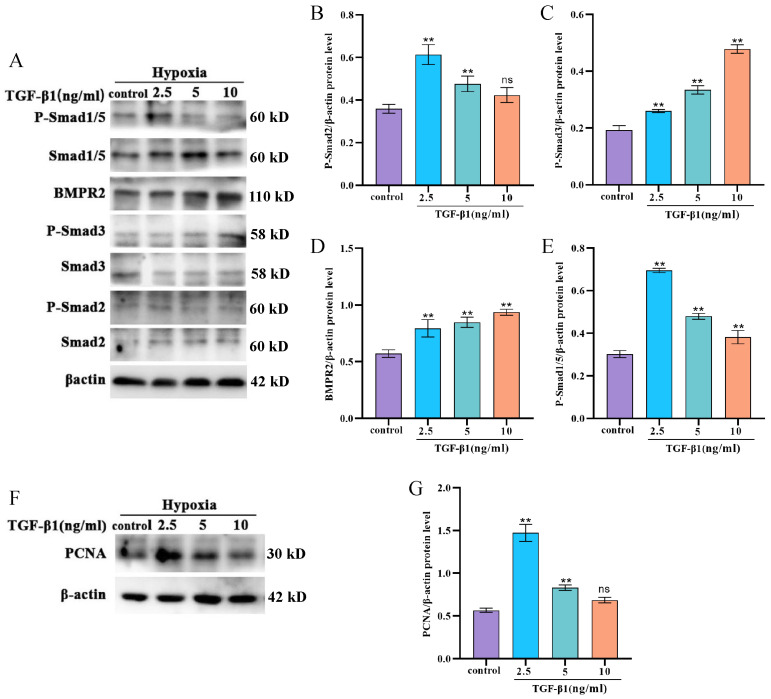
Effects of TGF-β1 treatment on the expression levels of TGF-β/BMP signaling pathway-related proteins and proliferation in yak PASMCs. (**A**–**E**) Western blot analysis of the expression levels of TGF-β/BMP signaling pathway-related proteins after treatment with various concentrations of TGFβ1 (2.5, 5, 10 ng/mL), with a densitometric analysis performed using ImageJ. (**F**,**G**) Western blot analysis of the expression levels of PCNA after treatment with various concentrations of TGFβ1 (2.5, 5, 10 ng/mL), with a densitometric analysis performed using ImageJ. ** p < 0.01, ns indicates insignificant differences.

The effects of various concentrations of TGF-β1 recombinant protein (2.5, 5, and 10 ng/mL) under hypoxic conditions on the proliferation of yak PASMCs were assessed. Western blotting indicated that TGF-β1 increased the phosphorylation levels of Smad2 and Smad3, downstream components of the TGF-β signaling pathway (Figure 4A–C). Notably, TGF-β1 also activated the expression of BMPR2 protein and the phosphorylation of Smad1/5 in the BMP signaling pathway (Figure 4D,E), suggesting that the addition of TGF-β1 under hypoxic conditions activates the BMP signaling pathway in yak PASMCs. In addition, the addition of TGF-β1 increased PCNA protein levels, thereby promoting the proliferation of yak PASMCs (Figure 4F,G).

### 3.5. Inhibition of the BMP Signaling Pathway Increases the Proliferation of Yak PASMCs

**Figure 5:**
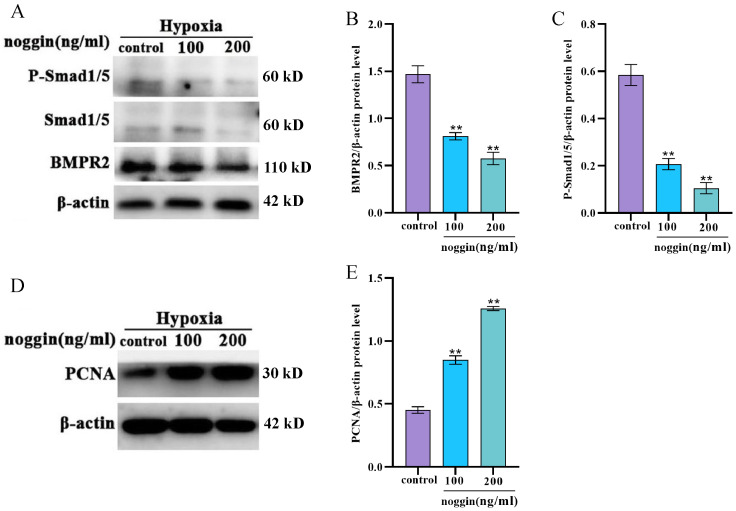
Effects of noggin treatment on BMP signaling pathway-related protein expression and proliferation in yak PASMCs. (**A**–**C**) Western blot analysis of the expression levels of BMP signaling pathway-related proteins after treatment with various concentrations of noggin (100 and 200 ng/mL), with a densitometric analysis performed using ImageJ. (**D**,**E**) Western blot analysis of the expression level of PCNA after treatment with various concentrations of noggin (100 and 200 ng/mL), with a densitometric analysis performed using ImageJ. ** p < 0.01.

To evaluate the role of the BMP signaling pathway in the proliferation of yak PASMCs under hypoxic conditions, the effects of noggin, an antagonist of the BMP signaling pathway, were assessed. Under hypoxic conditions, various concentrations of noggin (100 and 200 ng/mL) were added and cultured for 24 h. Western blot was used to measure expression levels of BMPR2 and phosphorylation levels of downstream Smad1/5. Noggin treatment significantly inhibited the expression of BMPR2 and P-Smad1/Smad5 (Figure 5A–C). In addition, as determined by Western blotting, noggin treatment significantly increased the proliferation of yak PASMCs (Figure 5D,E). These findings suggest that under hypoxic conditions, the noggin-mediated inhibition of the BMP signaling pathway significantly promotes the proliferation of yak PASMCs.

### 3.6. TGF-β1 and Noggin Promote the Migration of Yak PASMCs

**Figure 6:**
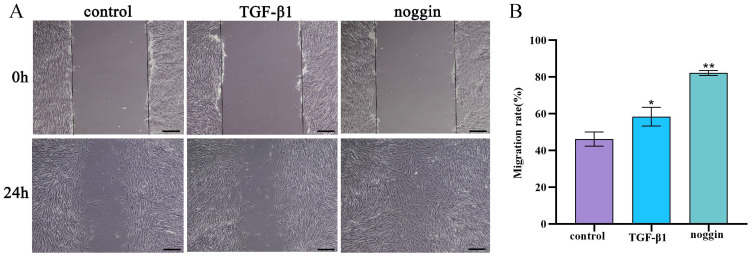
Effects of TGF-β1 and noggin on the migration of yak PASMCs. (**A**) Cell scratch assay showing the impact of TGF-β1 and a noggin inhibitor on the migration of yak PASMCs. bar = 100 μm. (**B**) Statistical analysis of cell migration rates. ** *p* < 0.01, * 0.01 < *p* < 0.05.

Yak PASMCs were seeded in 6-well plates; after creating a cell scratch, 5 ng/mL TGF-β1 recombinant protein and 100 ng/mL noggin inhibitor were added under hypoxic conditions and incubated for 24 h to assess PASMC migration. Both exogenous TGF-β1 and the noggin inhibitor promoted the migration of yak PASMCs significantly under hypoxic conditions (Figure 6A,B).

## 4. Discussion

The initial response to hypoxic conditions is hypoxic pulmonary vasoconstriction, which leads to vascular remodeling and a sustained increase in vascular resistance, ultimately progressing to pulmonary hypertension. In severe cases, this can cause right heart failure and death [14,15]. Therefore, hypoxia induction is commonly used to construct models of pulmonary hypertension. Smooth muscle cells, because of their inherent phenotypic structural characteristics, are the primary sites of vascular remodeling in response to hypoxia [15]. Under physiological conditions, a proper balance between the proliferation and apoptosis of vascular smooth muscle cells is essential for maintaining an appropriate vascular wall thickness [16,17]. Prolonged hypoxic exposure does not cause pathological damage to the yak lungs, including the development of pulmonary hypertension. At the cellular level, this can be interpreted as a good balance between the proliferation and apoptosis of yak pulmonary vascular smooth muscle cells under hypoxic stimuli, with the degree of vascular remodeling being within physiological limits. HIF-1α and HIF-2α are two early markers in the pulmonary circulation’s response to hypoxic stimuli. They initiate adaptative responses to hypoxia by inducing or inhibiting the regulation of vascular cell proliferation and apoptosis [18,19]. In the present study, the expression levels of HIF-1α and HIF-2α in yak PASMCs increased significantly in response to hypoxia and increased with the duration of exposure, supporting the reliability of the yak PASMC response to hypoxia.

Crosstalk between the TGF-β and BMP signaling pathways plays a critical role in maintaining a balance of pulmonary vascular smooth muscle cells. In this study, we examined the expression levels of BMP/Smad1/5 and TGF-β1/Smad2/3 signaling molecules in yak pulmonary artery smooth muscle cells under hypoxic conditions. We found that TGF-β signaling decreased with the upregulation of HIF-1α after 12 h or 24 h of hypoxic stimulation. However, this trend gradually reversed with increased exposure to hypoxia, differing from previous results for other animal models, where hypoxia-induced HIF-1α upregulation significantly activated TGF-β/Smad signaling, thereby promoting cell proliferation [20,21]. Of note, after 12 h and 24 h of continuous exposure to hypoxia, the expression levels of BMPRII and P-Smad1/5 were significantly higher than those in the normoxic group, and this increase in BMP signaling was gradually attenuated with prolonged hypoxia. These results were also different from those of previous studies of hypoxia-induced PH models, other PH models, and patients [22,23]. For example, significant increases in TGF-β1 and P-Smad3 levels have been detected in a rat model of pulmonary hypertension induced by hypoxia [24], and the significant downregulation of BMP/Smad1/5 and upregulation of TGF-β/Smad2/3 after 24 h of hypoxic treatment has been reported in primary cultured rat PASMCs [25]. Increasing research indicates that there is complex crosstalk between the TGF-β/Smad2/3 and BMP/Smad1/5 signaling pathways, with evidence for functional antagonism. The TGF-β signaling pathway promotes PASMC proliferation through the phosphorylation of downstream Smad2 and Smad3, while the BMP signaling pathway inhibits PASMC proliferation, conferring a protective effect on the vascular wall, and thus is a candidate target for clinical PH treatment. For example, the inhibition of the TGF-β1/Smad2/Smad3 signaling pathway using ML133 reduces the proliferation and migration of human PASMCs [26], and restoring the TGF-BMP balance in PAH models improves vascular remodeling [27]. Therefore, the lack of HPH in yaks exposed to hypoxia for long periods could potentially be explained by the activation of the BMP signaling pathway and the transient inhibition of TGF-β signaling under hypoxia in PASMCs.

Previous research has shown that TGF-β1 promotes the proliferation and migration of PASMCs. In this study, treating yak PASMCs with 2.5 ng/mL TGF-β1 under hypoxic conditions increased the phosphorylation levels of Smad2 and Smad3 significantly. The cell proliferation marker PCNA and cell scratch assay results consistently indicated that exogenous TGF-β1 significantly promoted the proliferation and migration of yak PASMCs. The observed response of yak PASMCs to exogenous TGF-β1 under hypoxic conditions was similar to those observed in other hypoxia-induced experimental animals, showing that co-stimulation of rat lung fibroblasts with hypoxia and TGF-β1 significantly increases cell proliferation [28]. In addition, past studies have also shown that TGF-β1 stimulation is associated with BMP signaling in a cell-specific manner. For instance, after treating adult lung-cultured PASMCs with TGF-β1 for 1 h, phosphorylated Smad1/5 expression significantly increased; however, this cross-stimulation was not detected in the lungs of newborns. The results of the present study suggest that the treatment of yak PASMCs with exogenous TGF-β1 can cross-stimulate BMP R-Smad1/5 signaling, leading to increases in BMPII expression and the phosphorylation of Smad1/5. The role of TGF-β1-stimulated BMP R-Smad signaling in PASMCs is still uncertain. The phosphorylation of Smad1/5 is mediated by BMP; however, recent studies suggest that TGF-β1-mediated BMP-R-Smad signaling may control genes involved in PASMC proliferation [28]. In summary, our results suggest that the substantial increase in BMPRII/Smad1/5 signaling in response to hypoxic conditions in yak PASMCs has an inhibitory effect on hypoxia-induced proliferation, thereby balancing TGF-β1/Smad2/3 signaling under hypoxic conditions and conferring a protective effect on yak PASMCs. The mechanism by which yak PASMCs activate BMP signaling to increase the phosphorylation of Smad1/5 under hypoxia is not clear and is worth further investigation.

In many patients with hereditary familial PAH, heterozygous mutations in BMPRII have been identified. Additionally, reductions in BMPRII protein levels and dysfunction in BMP signaling have also been noted in other forms of PAH and pulmonary diseases caused by vascular remodeling [29]. BMP signaling, which regulates the proliferation and apoptosis of various vascular resident cells, is essential in PASMCs [30]. Noggin, which shares a cysteine-knot ligand structure with BMP, acts as a classic inhibitor of BMP signaling and specifically binds to BMP-2, BMP-4, BMP-7, and BMP-14 but not to other types of BMPs [31,32]. In this study, we found that treatment with noggin (100 and 200 ng/mL) inhibited the expression of BMPRII and P-Smad1/5 significantly in yak PASMCs. Interestingly, we also observed that noggin treatment increased the expression of the cell proliferation marker protein PCNA significantly, suggesting that inhibiting the BMP/Smad1/5 signaling pathway can promote yak PASMC proliferation. Coupled with previous findings that BMP signaling pathway activity increased significantly after 12 h and 24 h of hypoxic induction in yak PASMCs, this further suggests that yak PASMCs balance hypoxia-induced proliferation by activating the BMP/Smad1/5 signaling pathway.

Previous studies of rat PAH models have shown that a reduction in BMP/Smad1/5 signaling is accompanied by an increase in TGF-β1/Smad2/3 signaling, and the TGF-β signaling inhibitor ALK5 attenuates these changes, effectively preventing the progression of PAH [33]. Moreover, some studies suggest that a reduction in BMPR2 signaling leads to the disruption of TGF-β/BMP signaling, facilitating the activation of TGF-β signaling and thus promoting cell proliferation [34]. This is consistent with our findings in yak PASMCs, showing that noggin, a typical inhibitor of BMP signaling, suppresses the BMP signaling pathway and downregulates the phosphorylation levels of Smad1/5, promoting the proliferation of yak PASMCs substantially. This increased proliferation may be achieved by converting the downregulation of BMP signaling into the upregulation of the TGF-β signaling pathway. The mechanism underlying this conversion and crosstalk between BMP and TGF-β is highly complex. Recent studies suggest that peroxisome proliferator-activated receptor (PPAR) serves as a bridge in this conversion. PPAR, a downstream effector of BMP2, interacts with the pro-proliferative TGF-β1-STAT3-FOX01 axis in vascular smooth muscle cells, thus inhibiting TGF-β signal-induced vascular remodeling [35].

## 5. Conclusions and Prospect

**Figure 7:**
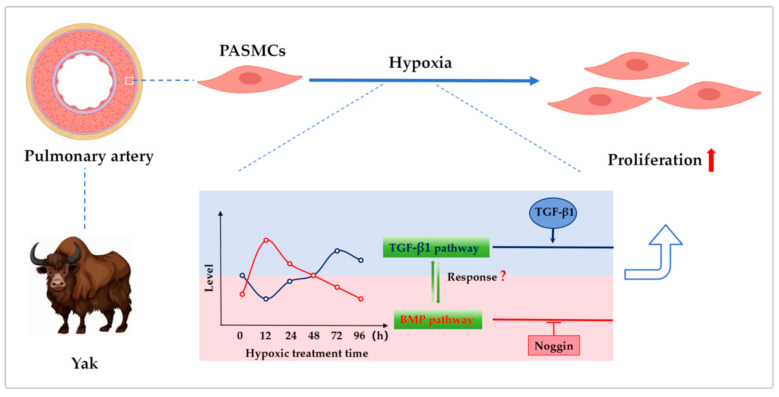
Schematic representation of the TGFβ/BMP signaling pathway crosstalk promoting proliferation of pulmonary artery smooth muscle cells in yaks under hypoxic conditions.

In summary, the results revealed that under hypoxic conditions, the dynamic regulation of the TGF-β/BMP signaling pathways promotes the proliferation of yak PASMCs (Figure 7).

This study has certain limitations. We investigated the impact of the TGFβ/BMP signaling pathway on PASMC proliferation in yaks under hypoxic conditions. However, it should be noted that the TGFβ and BMP signaling pathways may exhibit species-specific and cell-type-specific roles. Therefore, further research is required to determine whether the findings from this study can be generalized to other bovine breeds.

## Data Availability

All data generated during the current study are included in this manuscript.
